# LncRNA LINC00205 stimulates osteoporosis and contributes to spinal fracture through the regulation of the miR-26b-5p/KMT2C axis

**DOI:** 10.1186/s12891-023-06136-z

**Published:** 2023-04-04

**Authors:** Hongtao Wang, Weilin Xu, Xiaoqing Chen, Xiongfeng Mei, Zhonghua Guo, Juan Zhang

**Affiliations:** 1 Department of Rehabilitation Medicine, People’s Hospital of Dongxihu District, No. 48 Jinbei 1St Road, Jinghe Street, Dongxihu District, Wuhan, 430040 Hubei China; 2 Department of Orthopaedics, People’s Hospital of Dongxihu District, Wuhan, 430040 Hubei China

**Keywords:** lncRNA LINC00205, Osteoporosis, Spinal fracture, miR-26b-5p, KMT2C, Human mesenchymal stem cells

## Abstract

**Background:**

Osteoporosis (OP) is a common bone disease marked by decreased bone strength. Increasing evidence suggests that long non-coding RNA (lncRNAs) play important roles in the occurrence and progression of OP. This study aimed to investigate the role and mechanism of LINC00205 in the osteogenic differentiation of human mesenchymal stem cells (hMSCs) and OP.

**Methods:**

Bone tissue samples were obtained from healthy controls and patients with osteoporosis with a spinal fracture (OP-Frx) or without a spinal fracture (OP-no-Frx). HMSCs were cultured and induced to undergo osteogenic differentiation. The expression of LINC00205, lysine (K)-specific methyltransferase 2C (KMT2C), and miR-26b-5p in bone tissues and cells was evaluated using western blotting and real-time quantitative reverse transcription polymerase chain reaction (qRT-PCR). The effects of LINC00205, miR-26b-5p, and KMT2C on calcium deposition, alkaline phosphatase (ALP) activity, and mRNA levels of the osteogenic differentiation marker genes [ALP, osteocalcin (OCN), and runt-related transcription factor 2 (RUNX2)] were investigated using alizarin red S staining, an ALP activity assay, and qRT-PCR, respectively. Dual-luciferase reporter assay was performed to ascertain the binding relationship between miR-26b-5p and LINC00205/KMT2C.

**Results:**

LINC00205 and KMT2C were upregulated in patients with OP-Frx and OP-no-Frx, whereas miR-26b-5p was downregulated. Furthermore, LINC00205 and KMT2C expression decreased, whereas that of miR-26b-5p increased over time from day 7 to 21 of the osteogenic differentiation of hMSCs. The knockdown of LINC00205 and KMT2C significantly increased ALP activity, calcium deposition, and the expression of RUNX2, ALP, and OCN. In contrast, the inhibition of miR-26b-5p yielded the opposite result. These data suggest that LINC00205 inhibits the osteogenic differentiation of hMSCs by modulating the miR-26b-5p/KMT2C signaling axis.

**Conclusion:**

LINC00205 promotes OP and is involved in spinal fractures. LINC00205 is also a potential negative regulator of the osteogenic differentiation of hMSCs.

**Supplementary Information:**

The online version contains supplementary material available at 10.1186/s12891-023-06136-z.

## Introduction

Osteoporosis (OP) is one of the most prevalent bone diseases [1]. It is characterized by reduced bone strength and puts a person at a 40% lifetime risk of fractures [1]. Studies have shown that fractures in patients with OP most commonly occur in the wrist, hip, or spine [2]. Furthermore, osteoporotic spinal fractures significantly increase the mortality rate for 12 months and increase the risk of other medical complications [3, 4]. OP is becoming a heavy burden on healthcare systems worldwide, and its medical and socioeconomic implications will further increase with the aging population [5].

Long non-coding RNAs (lncRNAs) are non-protein coding RNAs transcribed by eukaryotic genomes [6]. They are involved in mRNA processing, maturation, and stability by acting as microRNA (miRNA) sponges or competing for binding sites on mRNAs [6]. In addition, lncRNAs are involved in various cellular activities and pathogenesis, including OP pathogenesis [7]. For example, plasma lncRNA NEF is downregulated in postmenopausal OP and is associated with long treatment cycles and high relapse rates [8]. BCAR4, another lncRNA, is downregulated during human mesenchymal stem cell (hMSC) differentiation and mitigates the progression of OP [9]. LINC00205, a newly studied lncRNA, has been found to act as a carcinogen in lung cancer, liver cancer, retinoblastoma, and other cancers by promoting the malignant proliferation of cancer cells [10–12]. However, the role of LINC00205 in OP remains to be elucidated.

The miRNAs are a class of non-coding RNAs (ncRNAs) that are known to degrade mRNA or inhibit its translation and consequently regulate gene expression [13–15]. Accumulating evidence indicates that some miRNAs, including miR-205-5p, miR-151a-3p, and miR-16a-5p, are involved in the development of OP [13–15]. They can regulate osteoblast and osteoclast differentiations to participate in osteogenesis and osteoclastogenesis, thereby leading to OP [16]. For example, miR-214 and miR-135-5p have been reported to regulate the proliferation and osteogenic differentiation of hMSCs [17, 18]. Another miRNA, miR-26b-5p, was found to be upregulated in hMSCs during osteogenesis and induce osteogenic differentiation [19].

Lysine (K)-specific methyltransferase 2C (KMT2C) is a histone methyltransferase involved in transcriptional co-activation. KMT2C is highly conserved and known to have evolved in unicellular eukaryotes [20]. Studies have shown that KMT2C is mutated in various diseases such as mental disabilities and cancer [21, 22]. A bioinformatics tool was used in this study to identify the possible binding sites between miR-26b-5p and KMT2C. The interaction of KMT2C and miR-26b-5p in the development of OP has not yet been studied or reported.

The aim of the present study was to look into the role and mechanism of action of LINC00205 in the progression of OP, as well as its role in regulating the osteogenic differentiation of human mesenchymal stem cells (hMSCs). We performed a comprehensive bioinformatics analysis to identify the target miRNA of LINC00205 and then established a lncRNA–miRNA-mRNA regulatory network. Based on the results of bioinformatics analysis, we hypothesized that LINC00205 might influence the progression of OP by regulating the miR-26b-5p/KMT2C axis. Therefore, we performed various rescue and functional experiments to investigate the role of the LINC00205/miR-26b-5p/KMT2C axis in regulating osteogenic differentiation of hMSCs and thus influencing OP progression.

## Materials and methods

### Human samples

Bone tissues from patients with OP (*N* = 24), divided into two groups, namely those with spinal fracture (OP-Frx) or without spinal fracture (OP-no-Frx), and from control subjects admitted to People’s Hospital of Dongxihu District were collected during hip arthroplasty. The patients in control group suffered from bone injury caused by external force such as sports, traffic accident, etc. This study was approved by the Research Ethics Committee of People’s Hospital of Dongxihu District. Written informed consent was obtained from each patient enrolled in the study. The inclusion criteria for patients with OP were a bone mineral density (BMD) of at least 2.5 standard deviations below the peak BMD (-2.5 T score) of healthy young women in the lumbar spine, total hip, or femoral neck after the age of 40 years. The exclusion criteria were a history of OP therapy, hormone replacement therapy, early menopause (< 40 years), abnormal menopause, acute gastrointestinal inflammation, or chronic renal failure. The clinical characteristics of the participants are summarized in Supplementary Table 1.

### Cell culture and osteoblast differentiation

HMSCs were obtained from the Chinese Academy of Sciences (Shanghai, China). They were maintained in α-modified Eagle's medium (α-MEM) supplemented with 10% fetal bovine serum (FBS; Gibco, Grand Island, NY, USA) and 1% penicillin–streptomycin (Thermo Fisher Scientific, Waltham, MA, USA) in an incubator at 37 ℃ with 5% CO_2._ To induce osteoblast mineralization, 100 nM dexamethasone, 10 mM β-glycerophosphate disodium salt hydrate, and 50 μg/mL L-ascorbic acid (Sigma-Aldrich, St. Louis, MO, USA) were added to the hMSC culture for 3 weeks [23]. Alizarin red S (ARS) staining and alkaline phosphatase (ALP) activity assay was performed to confirm successful osteoblast differentiation of hMSCs.

### Cell transfection

Small interfering RNAs (siRNAs) targeting LINC00205 (si-LINC00205), KMT2C (si-KMT2C), and negative control siRNAs (si-NC) were acquired from Ribobio (Guangzhou, China). The miR-26b-5p inhibitor, NC inhibitor, miR-26b-5p mimics, and NC mimics were sourced from SwitchGear Genomics (Menlo Park, CA, USA). HMSCs were transfected with 200 nM inhibitor, 100 nM mimic, or 75 nM siRNA using the Lipofectamine® 2000 Transfection Reagent (Thermo Fisher Scientific). The cells were collected 2 days post-transfection, and analyzed for transfection efficiency using qRT-PCR.

### qRT-PCR analysis

The RNeasy™ Mini Kit (QIAGEN, Hilden, Germany) was used to extract total RNA. The total RNA was then reverse transcribed into cDNA using the PrimeScript™ RT Kit (Takara, Tokyo, Japan). PCR was carried out in an ABI 7500 Real-Time PCR System (Applied Biosystems, Foster City, CA, USA) using the Takara SYBR® Green Master Mix. The expression levels of LINC00205, KMT2C, alkaline phosphatase (ALP), runt-related transcription factor 2 (RUNX2), and osteocalcin (OCN) were normalized to that of GAPDH using the delta-delta Ct (2^−∆∆Ct^) method [24].

The miRNA was isolated using miRNA Isolation Kit (Ambion, Austin, TX). Subsequently, miRNA was reverse transcribed into cDNA using the TaqMan miRNA Reverse Transcription Kit (Applied Biosystems) according to the manufacturer’s protocol. PCR was performed according to the standard TaqMan microRNA assay protocol. U6 served as an endogenous control for miR-26b-5p. All the primers used are listed in Table 1.

**Table 1:** Forward and reverse primers listed for real-time qPCR

Name	Sequence (5’-3’)
miR-26b-5p forward miR-26b-5p reverse LINC00205 forward LINC00205 reverse RUNX2 forward RUNX2 reverse ALP forward ALP reverse OCN forward OCN reverse KMT2C forward KMT2C reverse U6 forward U6 reverse GAPDH forward GAPDH reverse	TTCAAGTAATTCAGGATAGGT GTGCGTGTCGTGGAGTC GGCTTTTGTGCCTGGAAGTG GGGAAGTTCTGAGCTGGCAT CGGTAAAATCTGCGTGCTCT TTCCCTACGAGGATTTCAGC TGGACCTCATCAGCATTTGG GAGGGAAGGGTCAGTCAGGTT CCAGCGACTCTGAGTCTGACAA AACGGTGGTGCCATAGATGC GCAGAATCAAGGAGCTGTCTG TGCCTTGAGGTCAACGTACAATTG CTCGCTTCGGCAGCACA AACGCTTCACGAATTTGCGT CGCTAACATCAAATGGGGTG TTGCTGACAATCTTGAGGGAG

### Cell counting kit-8 (CCK-8) assay

In vitro cell proliferation was assessed using the CCK-8 Kit (Beyotime, Shanghai, China) according to the manufacturer’s instructions. Briefly, hMSCs were plated in 96-well plates at a density of 4 × 10^3^ cells/well, allowed to adhere to the dishes, and cultured for 24, 48, or 72 h. CCK-8 solution (10 μL) was pipetted into each well, and the cells were incubated further for 4 h. The absorbance of each well was measured at a wavelength of 450 nm using a microplate reader (Bio-Rad, Hercules, CA).

### Alkaline phosphatase (ALP) activity assay

HMSCs were collected, washed in phosphate-buffered saline, and fixed with pre-cooled 95% ethanol for 1 h. Subsequently, the cells were lysed with radioimmunoprecipitation assay (RIPA) buffer (Beyotime). The supernatant of the lysate was collected and the Alkaline Phosphatase Assay Kit (Beyotime) was used to determine the ALP activity. Substrates and p-nitrophenol were added to the collected supernatant, and the solution was incubated for 10 min at 37 ℃. The absorbance was quantified at a wavelength of 405 nm using a microplate reader (Bio-Rad).

### Alizarin red S (ARS) staining

HMSCs were fixed in paraformaldehyde (4%) at 25 ℃ for 30 min. Subsequently, the cells were stained with 0.1% ARS (Beijing Chemical Factory, Beijing, China) at 37 ℃ for 5 min. The stained cells were visualized and images were captured using an optical microscope (Olympus, Tokyo, Japan).

### Luciferase assay

LINC00205 and KMT2C wild-type sequences containing the predicted miR-26b-5p binding site were inserted into the psiCHECK2.0 vector (Promega, Madison, WA) and labeled as LINC00205-WT and KMT2C-WT, respectively. The corresponding mutant reporter vectors were labeled as LINC00205-MUT and KMT2C-MUT. HMSCs were transfected with 100 ng of reporter vector and 50 nM of miR-NC or miR-26b-5p mimic. The Dual-luciferase Reporter Assay System (Promega) was used to measure the luciferase activity 48 h after transfection.

### Western blot analysis

HMSCs were collected and lysed in RIPA buffer (Beyotime) for 30 min. The protein concentration was determined using a BCA Protein Assay Kit (Bio-Rad) and 20 µg of protein samples was resolved on a 10% gel by sodium dodecyl sulfate polyacrylamide gel electrophoresis (SDS-PAGE), followed by transfer to polyvinylidene fluoride (PVDF) membranes (Millipore, Billerica, MA). Subsequently, the membranes were blocked with 5% skimmed milk at 37 °C for 1 h. Next, they were incubated overnight at 4 °C with KMT2C (Cat #MA5-38,554, 1:2000, Invitrogen, Carlsbad, CA) or GAPDH (Cat #MA1-16,757, 1:2000, Invitrogen) antibodies. Thereafter, the membranes were incubated with a suitable HRP-conjugated secondary antibody (Cat # ab205719, 1:2000, Abcam, Cambridge, UK) at 25 ℃ for 1 h. Finally, the protein bands were visualized using an ECL substrate (Millipore). The intensity of the protein bands was analyzed using the Image Lab Software (Bio-Rad).

### Statistical analysis

Data from three independent experiments were analyzed using GraphPad Prism 7.0 (GraphPad Software, La Jolla, CA, USA) and are expressed as mean ± standard deviation (SD). The Shapiro–Wilk test was performed to assess whether the data had a normal distribution, and Fisher's F test/Brown-Forsythe test was done to check homogeneity of variance. Furthermore, Student’s *t*-test was used to assess the differences between two independent groups. One-way analysis of variance (ANOVA) with Tukey’s *post-hoc* test was used to evaluate differences among multiple groups. Statistical significance was set at *P* < 0.05.

## Results

### Knockdown of LINC00205 promotes osteogenic differentiation of hMSCs

**Fig. 1:**
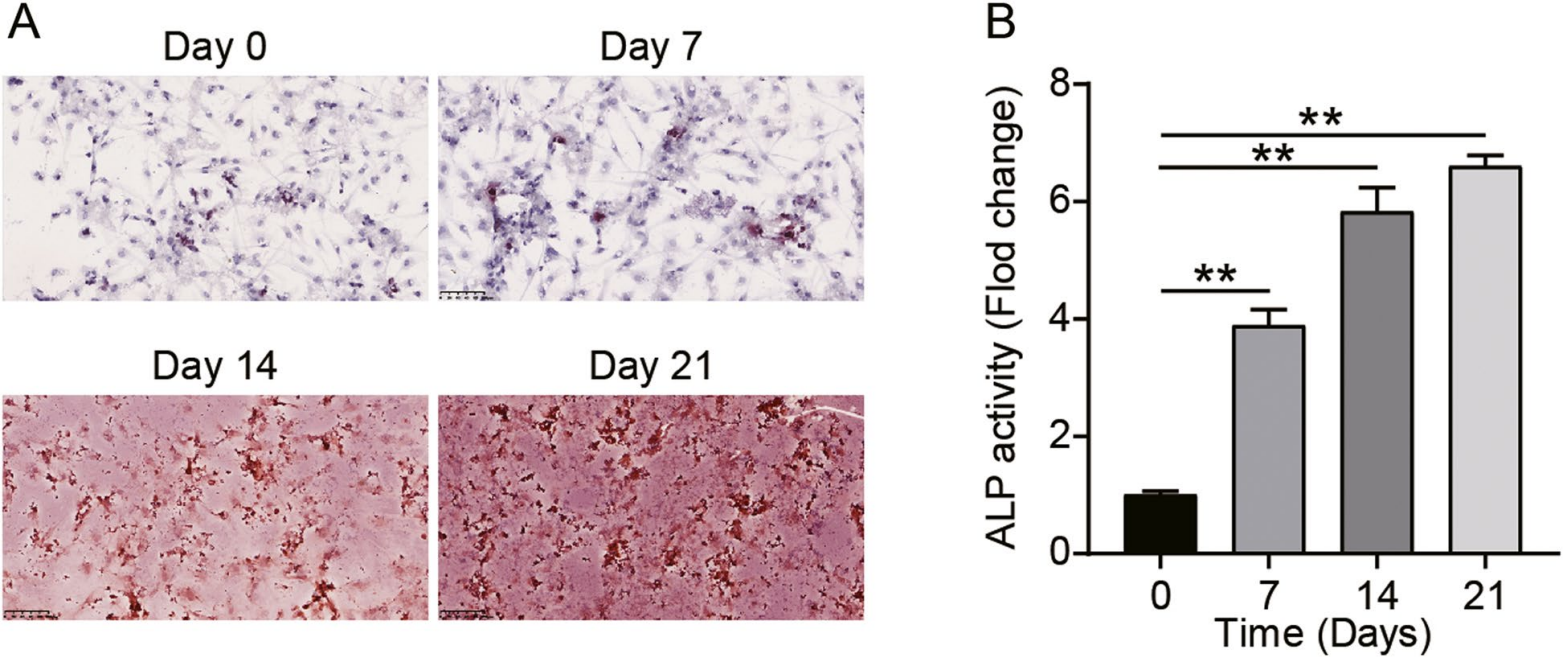
Confirmation of induction of osteoblastic differentiation of hMSCs. **A** Images of hMSCs stained with ARS on days 0, 7, 14, and 21 following treatment with the osteogenic medium. **B** The ALP enzymatic activity in hMSCs on days 0, 7, 14 and 21 following treatment with the osteogenic medium. **P < 0.001 when compared to day 0. All data were from three independent experiments

Before commencing any experiments, we confirmed the osteoblastic differentiation of hMSCs using the ARS staining and ALP activity assay. The ARS staining demonstrated successful osteogenic differentiation of hMSCs after 21 days as evidenced by a greater number of calcium nodules in hMSCs (Fig. 1A). The ALP enzymatic activity was measured as a fold of control and significantly increased on days 7, 14, and 21, with the highest activity being seen on the 21st day (Fig. 1B).

**Fig. 2:**
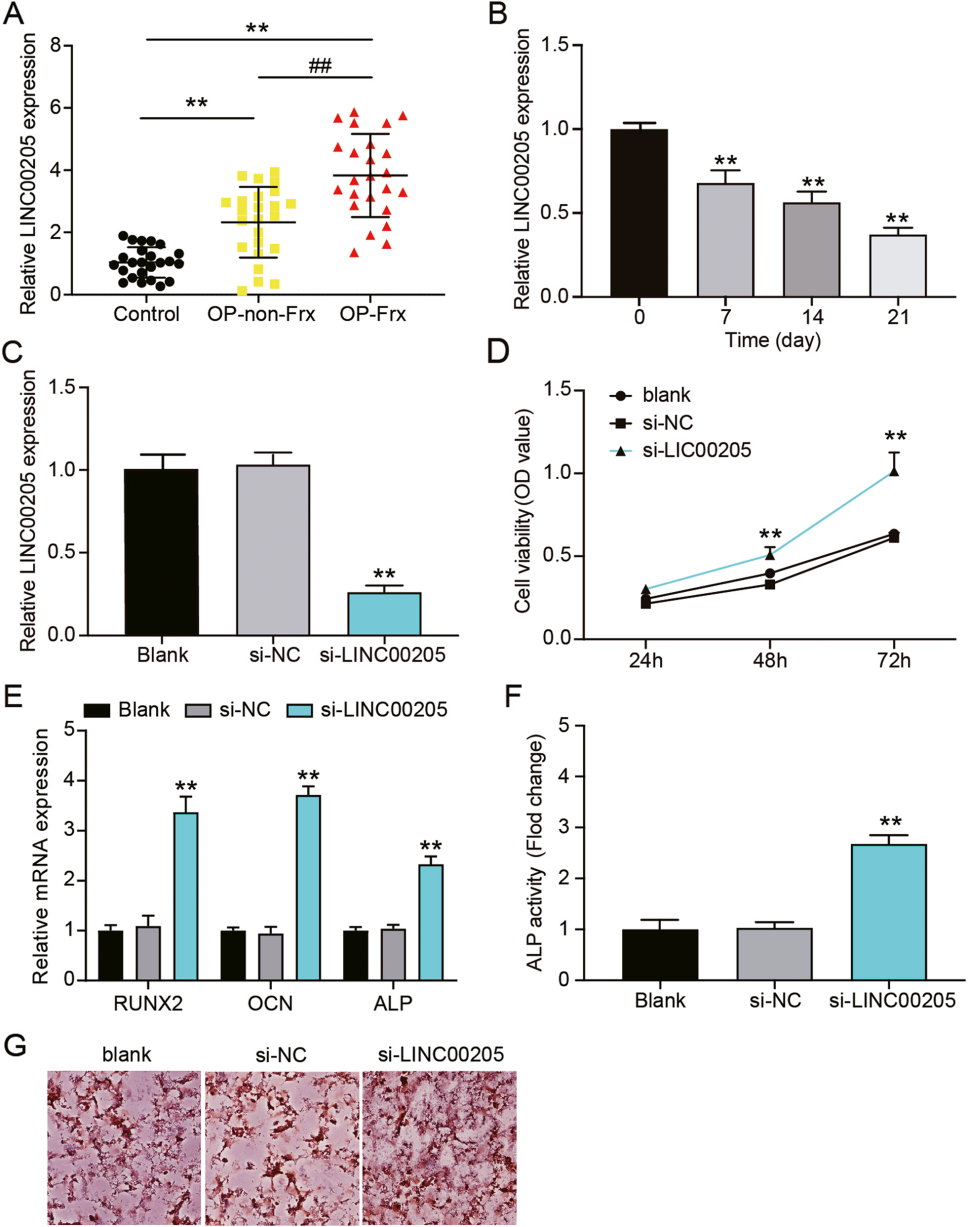
Knockdown of LINC00205 promoted osteogenic differentiation of hMSCs. **A** The relative expressions of LINC00205 in OP-Frx, OP-no-Frx, and healthy controls as quantified by qRT-PCR. **P < 0.001 and ##*P* < 0.001. **B** LINC00205 expressions at 7, 14, and 21 days of the osteoblast differentiation as analyzed by qRT-PCR. ** *P* < 0.001 when compared to day 0. **C** Expression levels of LINC00205 in hMSCs, quantified by qRT-PCR, after si-LINC00205 transfection. **P < 0.001 when compared to si-NC. **D** Viability of hMSCs treated with si-LINC00205 as assessed by the CCK-8 assay. **P < 0.001 when compared to si-NC at the same time. **E** Relative expression levels of RUNX2, OCN, and ALP mRNAs in hMSCs after treatment with si-LINC00205 were all analyzed using qRT-PCR. **P < 0.001 when compared to si-NC. **F** The ALP activity in hMSCs transfected with si-LINC00205 via the ALP activity assay. **P < 0.001 when compared to si-NC. **G** Images of si-LINC00205-transfected hMSCs stained with ARS. All data were from three independent experiments

The differential expression of LINC00205 in osteoporosis was assessed using qRT-PCR. The results demonstrated that LINC00205 expression was 1.2- and 3.9-fold higher in the bone tissues from patients with OP-no-Frx and OP-Frx, respectively, compared to that in control subjects in the validation cohort (Fig. 2A). Diagnostic potentials of levels of LINC00205 for OP-no-Frx were evaluated by receiver operating characteristic (ROC) curve analysis with patients with OP-no-Frx tissues as true positive cases and control tissues as true negative cases. Area under the curve was 0.825, with standard error of 0.06650 and 95% confidence interval (CI) of 0.6943–0.9550. Diagnostic potentials of levels of LINC00205 for OP-Frx were evaluated by ROC curve analysis with patients with OP-Frx tissues as true positive cases and OP-no-Frx tissues as true negative cases. Area under the curve was 0.797, with standard error of 0.06401 and 95% CI of 0.6714–0.9224 (Supplementary Fig. 1A). Moreover, changes in LINC00205 expression were observed during the osteogenic differentiation of hMSCs in vitro. Following osteogenic induction of hMSCs, LINC00205 levels decreased to 70%, 60%, and 40% on days 7, 14, and 21, respectively, of the original expression level on day 0 (Fig. 2B). Thus, LINC00205 expression was downregulated and the osteogenic differentiation period was prolonged, suggesting the involvement of LINC00205 in bone formation and that it may be associated with OP and vertebral fractures. Therefore, the role of LINC00205 in bone formation was investigated further.

HMSCs were transfected with si-LINC00205, and the levels of LINC00205 decreased by 75% when compared to si-NC transfected cells (Fig. 2C). Results from the CCK-8 assay revealed that the knockdown of LINC00205 enhanced the viability of the cells approximately 1.8-fold (Fig. 2D). The mRNA levels of RUNX2, OCN, and ALP were increased approximately 3.3-, 3.6-, and 2.5-fold, respectively, in the si-LINC00205 group compared to that in the si-NC group (Fig. 2E). Moreover, the reduced expression of LINC00205 resulted in increased ALP activity and greater number of calcium nodules in hMSCs (Fig. 2F and G). These results indicate that LINC00205 silencing positively modulates osteogenic differentiation in hMSCs.

### LINC00205 acts as a miR-26b-5p sponge

**Fig. 3:**
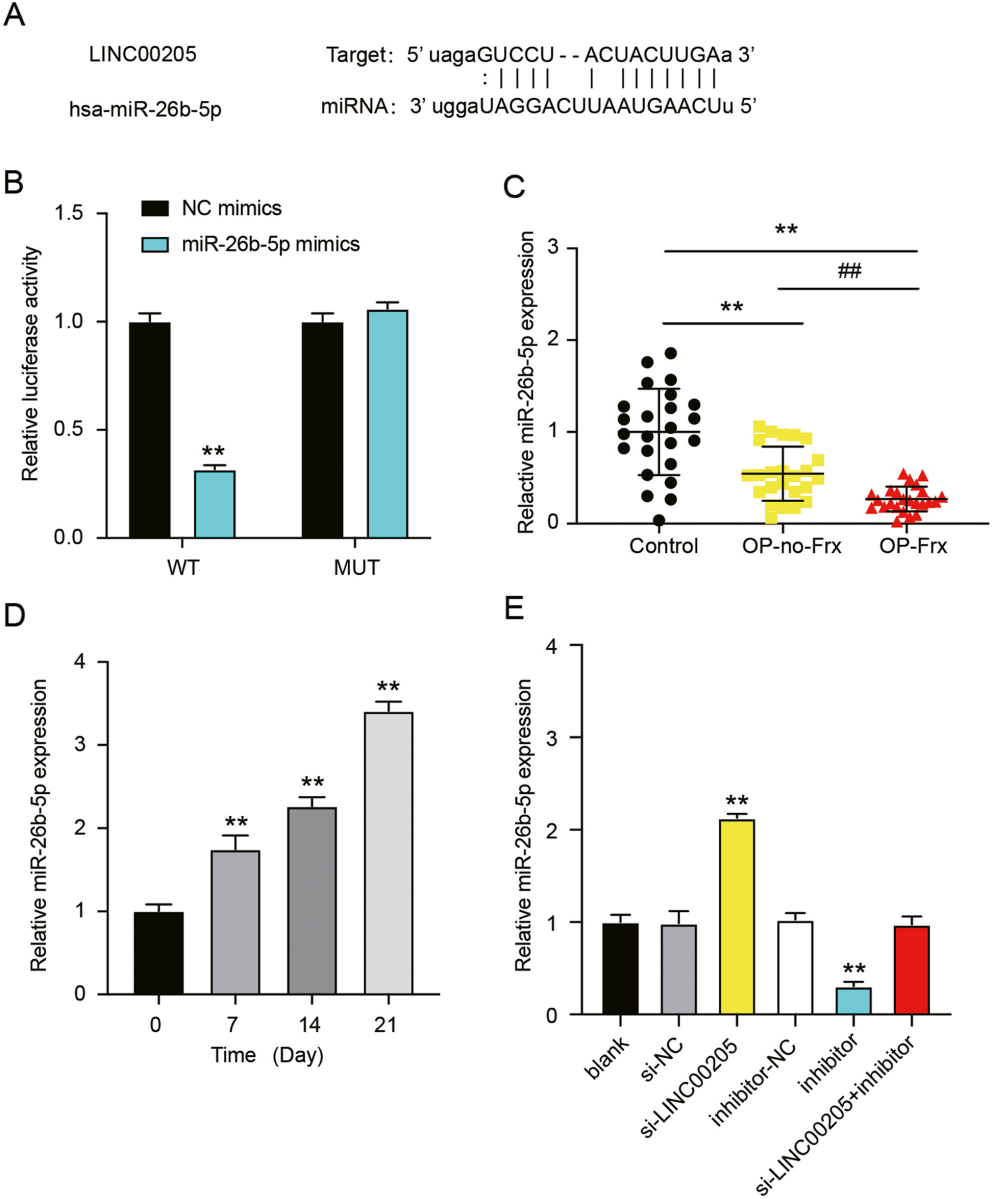
LINC00205 was a miR-26b-5p sponge. **A** MiR-26b-5p conserved seed sequence in LINC00205 identified using the tool starBase. **B** The relative luciferase activities of the hMSCs transfected with a NC-mimic or miR-26b-5p mimic plus a dual luciferase vector carrying the wild-type (WT) or mutant (MUT) LINC00205. The reduced luciferase activity confirms the binding between LINC00205 and miR-26b-5p. **P < 0.001 when compared to WT + NC-mimic. **C** The relative miR-26b-5p expressions among OP-Frx, OP-no-Frx, and healthy controls from the validation set were evaluated via qRT-PCR. **P < 0.001 and ## *P* < 0.001. **D** qRT-PCR test of miR-26b-5p expression at 7, 14, and 21 days of osteoblast differentiation. **P < 0.001 when compared to day 0. **E** Relative miR-26b-5p expressions among hMSCs after transfection were analyzed by qRT-PCR. A notable rise in miR-26b-5p levels was observed after transfection with si-LINC00205. Meanwhile the reverse was observed in miR-26b-5p inhibitor transfected cells. **P < 0.001 when compared to si-NC; ##*P* < 0.001 when compared to inhibitor-NC; and &&*P* < 0.001 when compared to si-LINC00205 + inhibitor. All data were from three independent experiments

The sequences of LINC00205 and miR-26b-5p were analyzed using starBase to elucidate the molecular mechanism through which LINC00205 regulates osteogenic differentiation. The binding sites of LINC00205 and miR-26b-5p are shown in Fig. 3A. To further verify that LINC00205 is a potential miR-26b-5p sponge, a dual-luciferase reporter system with WT and mutant LINC00205 was constructed. The luciferase activity remained almost unchanged following co-transfection of the mutant LINC00205 and miR-26b-5p mimic. In contrast, a 70% reduction in luciferase activity was observed in cells co-transfected with the LINC00205-WT and miR-26b-5p mimic (Fig. 3B). Additionally, miR-26b-5p levels were measured in clinical samples, and qRT-PCR analysis showed a 60% and 80% decline in miR-26b-5p levels in the bone tissues of patients with OP-no-Frx and OP-Frx, respectively, compared to healthy subjects (Fig. 3C). Diagnostic potentials of levels of miR-26b-5p for OP-no-Frx were evaluated by ROC curve analysis with patients with OP-no-Frx tissues as true positive cases and control tissues as true negative cases. Area under the curve was 0.785, with standard error of 0.06816 and 95% CI of 0.6511–0.9183. Diagnostic potentials of levels of miR-26b-5p for OP-Frx were evaluated by ROC curve analysis with patients with OP-Frx tissues as true positive cases and OP-no-Frx tissues as true negative cases. Area under the curve was 0.787, with standard error of 0.06937 and 95% CI of 0.6505–0.9225 (Supplementary Fig. 1B). In addition, miR-26b-5p levels increased over time during the osteoblast differentiation of hMSCs (Fig. 3D). These results demonstrated an opposite trend between the expression of LINC00205 and miR-26b-5p during bone formation. Hence, we speculate that LINC00205 downregulates miR-26b-5p in hMSCs via its sponge effect. The co-knockdown of LINC00205 and miR-26b-5p in hMSCs revealed that si-LINC00205 treatment upregulated miR-26b-5p and that miR-26b-5p inhibitor reversed the effect of LINC00205 knockdown (Fig. 3E). These data indicate that miR-26b-5p expression is decreased in OP, and LINC00205 acts as a miR-26b-5p sponge.

### LINC00205 inhibits osteogenic differentiation of hMSCs by sponging miR-26b-5p

**Fig. 4:**
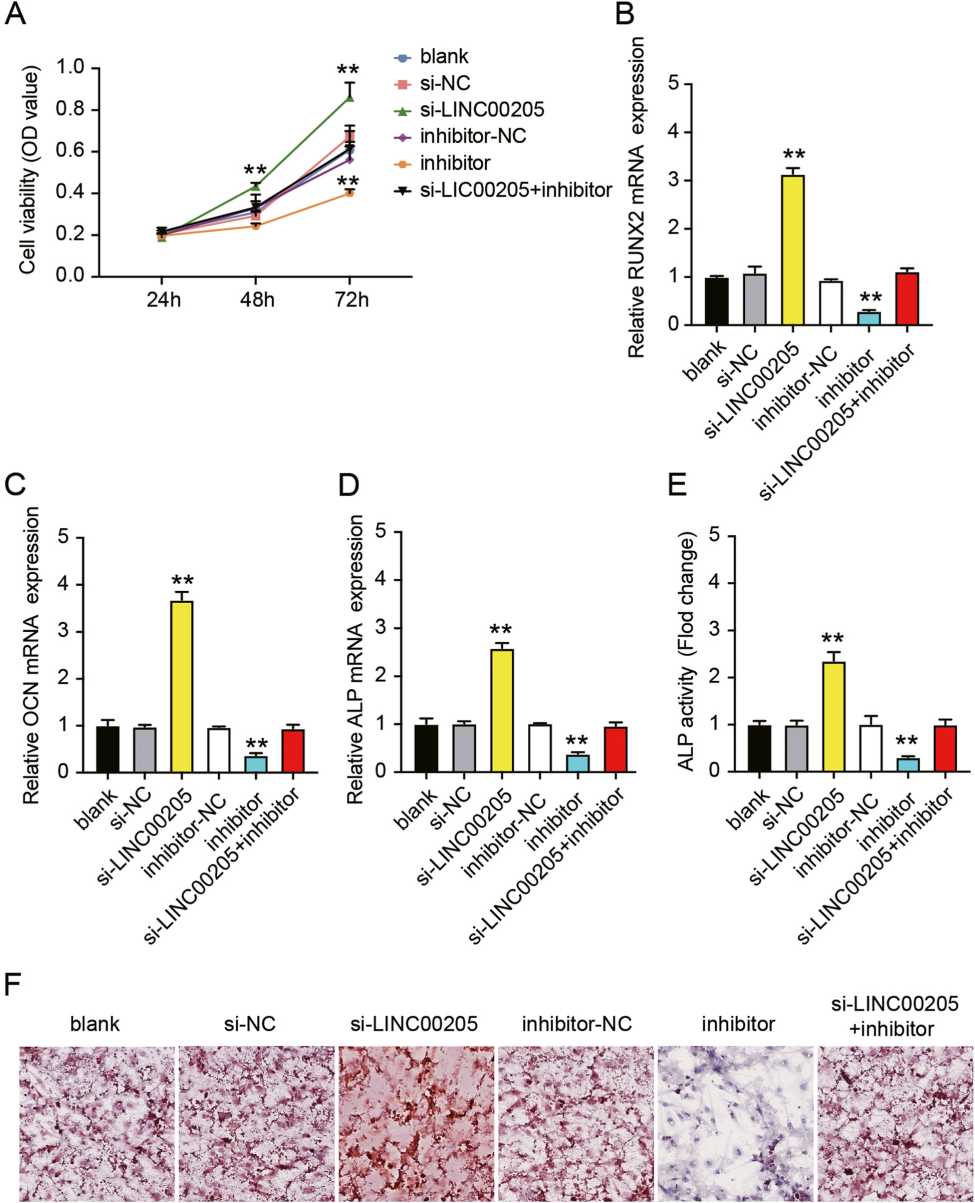
LINC00205 hindered the osteogenic differentiation of hMSCs by serving as miR-26b-5p sponge. **A** The CCK-8 assay revealed a lower viability in miR-26b-5p inhibitor transfected hMSCs compared to those that had si-LINC00205 transfection. **P < 0.001 when compared to si-NC; ##*P* < 0.001 when compared to inhibitor-NC; and &&*P* < 0.001 when compared to si-LINC00205 + inhibitor at the same time. **B**-**D** The relative expressions of RUNX2 (**B**), OCN (**C**), and ALP (**D**) mRNAs in hMSCs after si-LINC00205 or miR-26b-5p inhibitor transfection as quantified by qRT-PCR. The expressions of the three mRNAs are the highest among cells transfected with si-LINC00205, while the opposite is true for miR-26b-5p inhibitor transfected cells. **P < 0.001 when compared to si-NC; ##*P* < 0.001 when compared to inhibitor-NC; and &&*P* < 0.001 when compared to si-LINC00205 + inhibitor. **E** The ALP activity assay revealed that the ALP activity was the highest in hMSCs transfected with si-LINC00205, while was the lowest in hMSCs transfected with miR-26b-5p inhibitor. **P < 0.001 when compared to si-NC; ##*P* < 0.001 when compared to inhibitor-NC; and &&*P* < 0.001 when compared to si-LINC00205 + inhibitor. **F** Images of the si-LINC00205 and miR-26b-5p inhibitor transfected hMSCs stained with ARS. All data were from three independent experiments

Next, we sought to determine the roles of LINC00205 and miR-26b-5p in osteogenic differentiation of hMSCs. Transfection with a miR-26b-5p inhibitor significantly inhibited osteoblast differentiation. In miR-26b-5p inhibitor transfected cells, cell viability was reduced by approximately 28%, while RUNX2, OCN, and ALP levels were reduced by approximately 80%, 75%, and 70%, respectively, and ALP activity was reduced by approximately 85% (Fig. 4A-E). In addition, miR-26b-5p knockdown partly eliminated the si-LINC00205-mediated effects on osteogenic differentiation (Fig. 4A-E). ARS staining revealed that the miR-26b-5p inhibitor reduced calcium nodules in hMSCs and reversed the si-LINC00205-induced increase in calcium nodules (Fig. 4F). These results suggest that depletion of miR-26b-5p reverses the effect of LINC00205 silencing on the osteogenic differentiation of hMSCs.

### MiR-26b-5p targets KMT2C

**Fig. 5:**
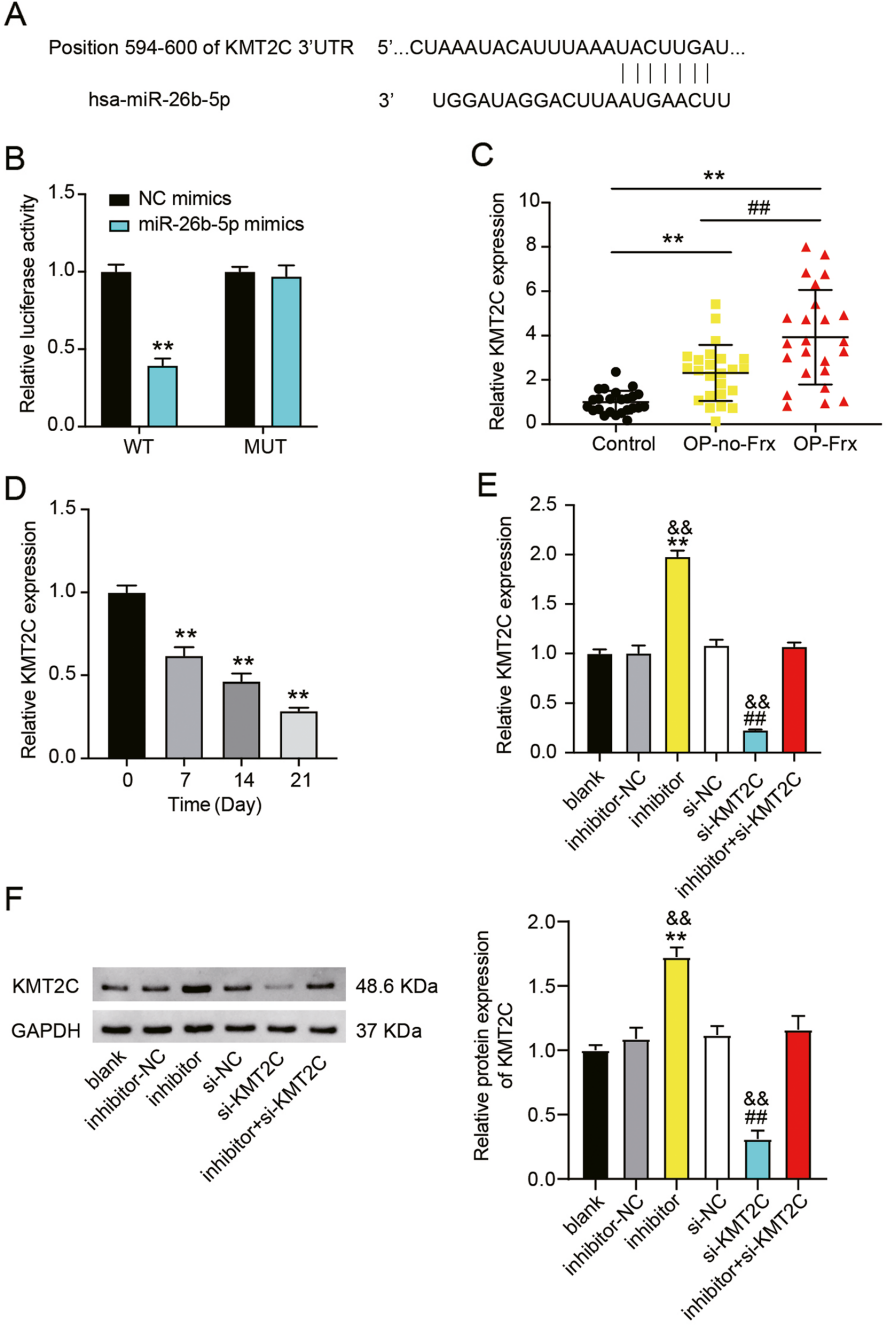
MiR-26b-5p targeted the KMT2C gene. **A** The miR-26b-5p conserved seed sequence in KMT2C as predicted by TargetScan. **B** The relative luciferase activities of hMSCs transfected with a NC-mimic or miR-26b-5p mimic plus a dual luciferase vector carrying the WT or MUT KMT2C. The reduced luciferase activity confirms the KMT2C and miR-26b-5p binding. **P < 0.001 when compared to WT + NC mimics. **C** Relative KMT2C expressions among OP-Frx, OP-no-Frx, and healthy controls from the validation set were evaluated via qRT-PCR. **P < 0.001 and ##*P* < 0.001. **D** KMT2C expressions analyzed by qRT-PCR at 7, 14, and 21 days of osteoblast differentiation. **P < 0.001 when compared to day 0. **E** Relative KMT2C mRNA expression in hMSCs after treatment with si-KMT2C or miR-26b-5p inhibitor, as analyzed by qRT-PCR. **P < 0.001 when compared to inhibitor-NC; ##*P* < 0.001 when compared to si-NC; and &&*P* < 0.001 when compared to inhibitor + si-KMT2C. **F** Protein bands (left) and relative protein expressions of KMT2C evaluated by Western blotting analysis of hMSCs after si-KMT2C or miR-26b-5p inhibitor transfection. **P < 0.001 when compared to inhibitor-NC; ##*P* < 0.001 when compared to si-NC; and &&*P* < 0.001 when compared to inhibitor + si-KMT2C. All data were from three independent experiments

Next, we sought to elucidate the downstream regulatory mechanism of miR-26b-5p. TargetScan analysis predicted the binding between miR-26b-5p and KMT2C (Fig. 5A). Furthermore, in luciferase assay, the co-treatment with the miR-26b-5p mimic and KMT2C-WT caused > 60% reduction in luciferase activity compared to co-transfection with the NC mimic and KMT2C-WT (Fig. 5B). Next, we analyzed the expression pattern of KMT2C in the clinical samples and found that KMT2C was higher in the bone tissues of patients with OP-Frx and OP-no-Frx than that in control subjects (Fig. 5C). Diagnostic potentials of levels of KMT2C for OP-no-Frx were evaluated by ROC curve analysis with patients with OP-no-Frx tissues as true positive cases and control tissues as true negative cases. Area under the curve was 0.833 with standard error of 0.06112 and 95% CI of 0.7135–0.9532. Diagnostic potentials of levels of KMT2C for OP-Frx were evaluated by ROC curve analysis with patients with OP-Frx tissues as true positive cases and OP-no-Frx tissues as true negative cases. Area under the curve was 0.734, with standard error of 0.07401 and 95% CI of 0.5293–0.8795 (Supplementary Fig. 1C). Moreover, KMT2C expression decreased during osteogenic differentiation of hMSCs (Fig. 5D). To determine the relationship between KMT2C and miR-26b-5p, hMSCs were transfected with si-KMT2C and miR-26b-5p inhibitors. The treatment with si-KMT2C was observed to reduce both the mRNA and protein levels of KMT2C, whereas the miR-26b-5p inhibitor restored KMT2C levels (Fig. 5E and F). These results indicate that KMT2C is abnormally expressed in OP and during osteogenic differentiation of hMSCs and that it is negatively regulated by miR-26b-5p.

### Silencing KMT2C weakens the suppressive effect of miR-26b-5p inhibitor on the osteogenic differentiation of hMSCs

**Fig. 6:**
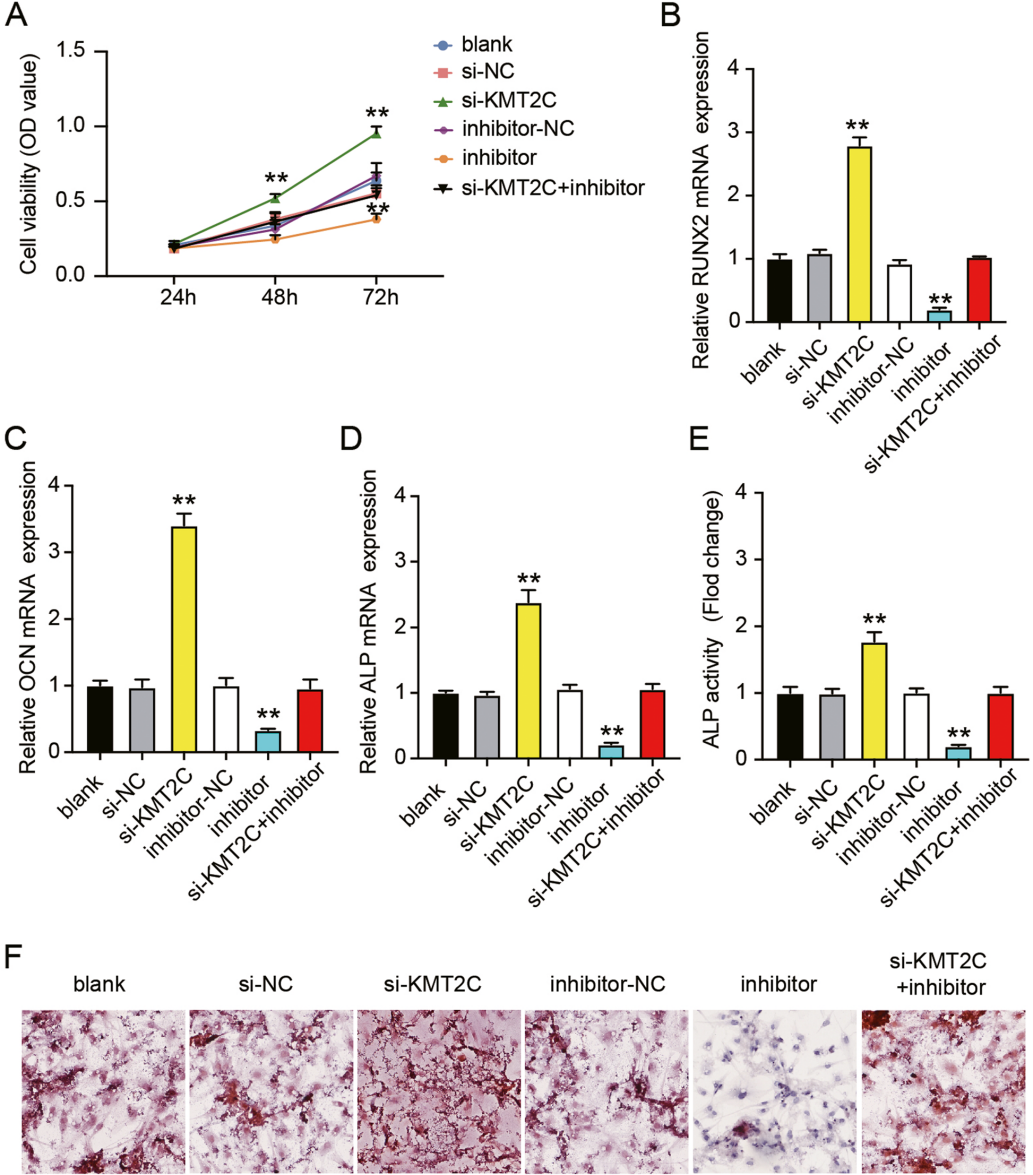
Silencing KMT2C attenuated the repressive effect of miR-26b-5p inhibitor on the osteogenic differentiation of hMSCs. **A** The CCK-8 assay demonstrated a lower viability in miR-26b-5p inhibitor transfected hMSCs, whereas a higher viability was observed after si-KMT2C transfection. **P < 0.001 when compared to si-NC; ##*P* < 0.001 when compared to inhibitor-NC; and &&*P* < 0.001 when compared to si-KMT2C + inhibitor at the same time. **B**-**D** The relative expressions of RUNX2 (**B**), OCN (**C**), and ALP (**D**) mRNAs in hMSCs after treatment with si-KMT2C or miR-26b-5p inhibitor as measured by qRT-PCR. **P < 0.001 when compared to si-NC; ##*P* < 0.001 when compared to inhibitor-NC; and &&*P* < 0.001 when compared to si-KMT2C + inhibitor. **E** The ALP activity assay revealed that the ALP activity was the highest in hMSCs transfected with si-KMT2C, while was the lowest with miR-26b-5p inhibitor transfection. **P < 0.001 when compared to si-NC; ##*P* < 0.001 when compared to inhibitor-NC; and &&*P* < 0.001 when compared to si-KMT2C + inhibitor. **F** Images of the si-KMT2C and miR-26b-5p inhibitor transfected hMSCs stained with ARS. All data were from three independent experiments

To investigate the effect of miR-26b-5p/KMT2C on osteogenic differentiation, we analyzed the viability of hMSCs using CCK-8 assay following miR-26b-5p or KMT2C knockdown. We observed that si-KMT2C increased cell viability by 1.4-fold, whereas miR-26b-5p inhibitor reversed the effect of si-KMT2C on cell viability (Fig. 6A). The qRT-PCR results demonstrated that silencing of KMT2C enhanced RUNX2, OCN, and ALP expression levels and restored the miR-26b-5p inhibitor-mediated downregulation of RUNX2, OCN, and ALP (Fig. 6B-D). ALP activity analysis showed that KMT2C knockdown promoted ALP activity in hMSCs and abolished the effect of miR-26b-5p silencing (Fig. 6E). In addition, ARS staining revealed that si-KMT2C promoted calcium nodule formation in hMSCs and reversed the effects of the miR-26b-5p inhibitor on calcium nodules (Fig. 6F). These data suggest that low KMT2C expression enhances the osteogenic differentiation of hMSCs and attenuates the repressive effect of miR-26b-5p inhibitor on osteogenic differentiation.

## Discussion

OP is a bone disorder caused by an imbalance in bone remodeling, which involves bone resorption and formation mediated by osteoclasts and osteoblasts, respectively [25]. Osteoblasts have a key role in bone formation and a new bone matrix is formed by the proliferation, differentiation, and matrix mineralization of pre-osteoblasts [26]. HMSCs are a source of osteoblasts and play a key role in bone tissue renewal [27]. ALP and OCN are established markers of late osteogenic differentiation [28]. RUNX2 has also been reported to be important for osteogenic differentiation and bone development [29, 30]. It regulates downstream molecules, including OCN, and is a crucial target in osteogenic differentiation [29, 30]. In this study, we looked into the role of the LINC00205/miR-26b-5p/KMT2C axis on regulating the osteogenic differentiation of hMSCs by examining ALP, OCN, RUNX2, and calcium deposition in them and subsequently the influence on the progression of OP.

LncRNAs regulate osteogenic differentiation; thus, they are important for the development of OP [31]. For example, Tang et al. showed that lncRNA IGF2-AS promotes osteogenic differentiation of hMSCs as evidenced by increased ARS staining and levels of ALP, osterix (Osx), OCN, and RUNX2 [32]. Several studies have revealed that LINC00205 is involved in the regulation of various biological functions in cells. Xie et al. [33] found that LINC00205 was elevated in lung cancer and that its knockdown inhibited the growth and migration of cancer cells and increased apoptosis. Similarly, Zhang et al. [12] showed that depletion of LINC00205 reduced the proliferation and metastasis of retinoblastoma cells and promoted apoptosis. We examined LINC00205 levels in OP-no-Frx and OP-Frx. Although LINC00205 was highly expressed in OP, its level was higher in OP-Frx suggesting that LINC00205 may worsen OP. Hence, we further looked into the role of LINC00205 in regulating the osteogenic differentiation of hMSCs. In vitro experiments showed that LINC00205 was under-expressed during osteogenic differentiation, and its knockdown promoted ALP, OCN, and RUNX2 expression, ALP activity, and calcium deposition. These results indicate the involvement of LINC00205 in the osteogenic differentiation of hMSCs.

The target-mimetic and sponge/decoy functions of lncRNAs have recently been uncovered [34]. Several reports have revealed the role of miR-26b-5p in bone diseases. MiR-26b-5p accelerates chondrocyte aging, cartilage degeneration, and osteoarthritis progression [35]. Furthermore, miR-26b-5p induces calcium deposition and promotes the expression of osteogenic genes such as ALP, OPN, OCN, and COL1A1 in rat bone marrow-derived mesenchymal stem cells (BMSCs) [19]. Because miR-26b-5p induces osteogenic differentiation, we analyzed its expression in OP in this study. The results revealed low expression of miR-26b-5p in OP-Frx and OP-no-Frx, and further knockdown inhibited the osteogenic differentiation of hMSCs. Furthermore, targeting analysis revealed that LINC00205 sponges miR-26b-5p. Rescue experiments demonstrated that miR-26b-5p interference reversed LINC00205 knockdown-induced osteogenic differentiation of hMSCs. This study is the first to reveal that LINC00205 regulates the osteogenic differentiation of hMSCs by sponging miR-26b-5p.

KMT2C promotes the synthesis of enhancer RNA and transcription promoters. It is also crucial in the activation of enhancers and cell type specific gene expression during cell differentiation [36, 37]. In addition, KMT2C may affect the enhancer activity of genes that influence invasive and metastatic properties in osteosarcoma [20, 38]. However, there are limited reports on the role of KMT2C in osteogenic differentiation and development of OP. Bioinformatics analysis revealed that miR-26b-5p targets and represses KMT2C expression. Moreover, KMT2C expression was examined in the bone tissues of patients with OP and in hMSCs during osteogenic differentiation. A higher level of KMT2C was observed in OP-no-Frx and OP-Frx patients compared to control subjects. The levels of KMT2C were also higher in hMSCs, which decreased with the progression of osteogenic differentiation. It has been suggested that KMT2C may be used to treat vertebral fractures and OP. This study revealed that silencing KMT2C promotes RUNX2, OCN, and ALP mRNA levels, ALP activity, and calcium nodule formation in hMSCs in vitro. Moreover, KMT2C partially attenuated miR-26b-5p interference-mediated inhibition of osteogenic differentiation. This suggests that miR-26b-5p directly targets KMT2C to regulate osteogenic differentiation of hMSCs.

Admittedly, this study has several limitations. In vivo experiments to investigate the crosstalk between LINC00205/miR-26b-5p/KMT2C were not carried out in this study. This issue needs to be addressed in future studies to further elucidate the effects of LINC00205 on OP and vertebral fractures in animals. In addition, the downstream mechanism of LINC00205/miR-26b-5p/KMT2C axis and the miR-26b gene cluster including miR-26b-3p merit further investigation.

In conclusion, this is the first report of the mechanism and expression of LINC00205/miR-26b-5p/KMT2C in OP and vertebral fractures. LINC00205 may worsen OP by reducing hMSCs viability, levels of osteogenic differentiation marker genes, and calcium nodule formation. LINC00205 inhibits osteogenic differentiation of hMSCs by acting on the miR-26b-5p/KMT2C axis. This finding may also be associated with vertebral fractures. The results of this study revealed the mechanism of LINC00205 in the development of OP. This study also provides new insights and perspectives for improving OP treatment.

## Supplementary Information

**Additional file 1: Supplementary Table 1.** Clinical characteristics of participants.

**Additional file 2: Supplementary Figure 1**

**Additional file 3.**

## Data Availability

The datasets that have been used and/or analyzed during the study are available from the corresponding author upon reasonable request.
