# Supplementary material for: LncRNA LINC00205 stimulates osteoporosis and contributes to spinal fracture through the regulation of the miR-26b-5p/KMT2C axis
Source: BMC Musculoskelet Disord. 2023 Apr 4;24:262. doi: 10.1186/s12891-023-06136-z (PMC10071705; doi:10.1186/s12891-023-06136-z)
Supplement: Supplementary file 1 — Additional file 1: Supplementary Table 1. Clinical characteristics of participants. [file 12891_2023_6136_MOESM1_ESM.docx]

Supplementary Table 1. Clinical characteristics of participants.

| Variable | Control (n = 24) | OP-no-Frx (n = 24) | OP-Frx (n = 24) |
| --- | --- | --- | --- |
| Sex, no. (%) |  |  |  |
| Female | 11 (45.83) | 18 (75.00) | 16 (66.67) |
| Male | 13 (54.17) | 6 (24.00) | 8 (33.33) |
| Age (y) | 55.67 ± 14.31 | 61.38 ± 13.64 | 77.25 ± 8.41 |
| Weight (kg) | 60.33 ± 19.51 | 57.25 ± 10.44 | 53.62 ± 11.75 |
| Height (cm) | 161.42 ± 11.37 | 159.15 ± 10.26 | 157.22 ± 8.48 |
| BMI (kg/m^2^) | 24.79 ± 2.53 | 23.28 ± 2.09 | 22.07 ± 3.62 |
| Spine BMD (g/cm^2^) | 1.217 ± 0.06 | 0.851 ± 0.005 | 0.716 ± 0.006 |
| Spine Z-score | 1.240 ± 0.528 | -1.719 ± 0.464 | -3.531 ± 0.602 |
| Spine T-score | 0.261 ± 0.107 | -1.722 ± 0.607 | -3.307 ± 0.719 |

BMI, Body mass index; BMD, Bone mineral density.
