# Supplementary figures and images for: LncRNA LINC00205 stimulates osteoporosis and contributes to spinal fracture through the regulation of the miR-26b-5p/KMT2C axis
Source: BMC Musculoskelet Disord. 2023 Apr 4;24:262. doi: 10.1186/s12891-023-06136-z (PMC10071705; doi:10.1186/s12891-023-06136-z)

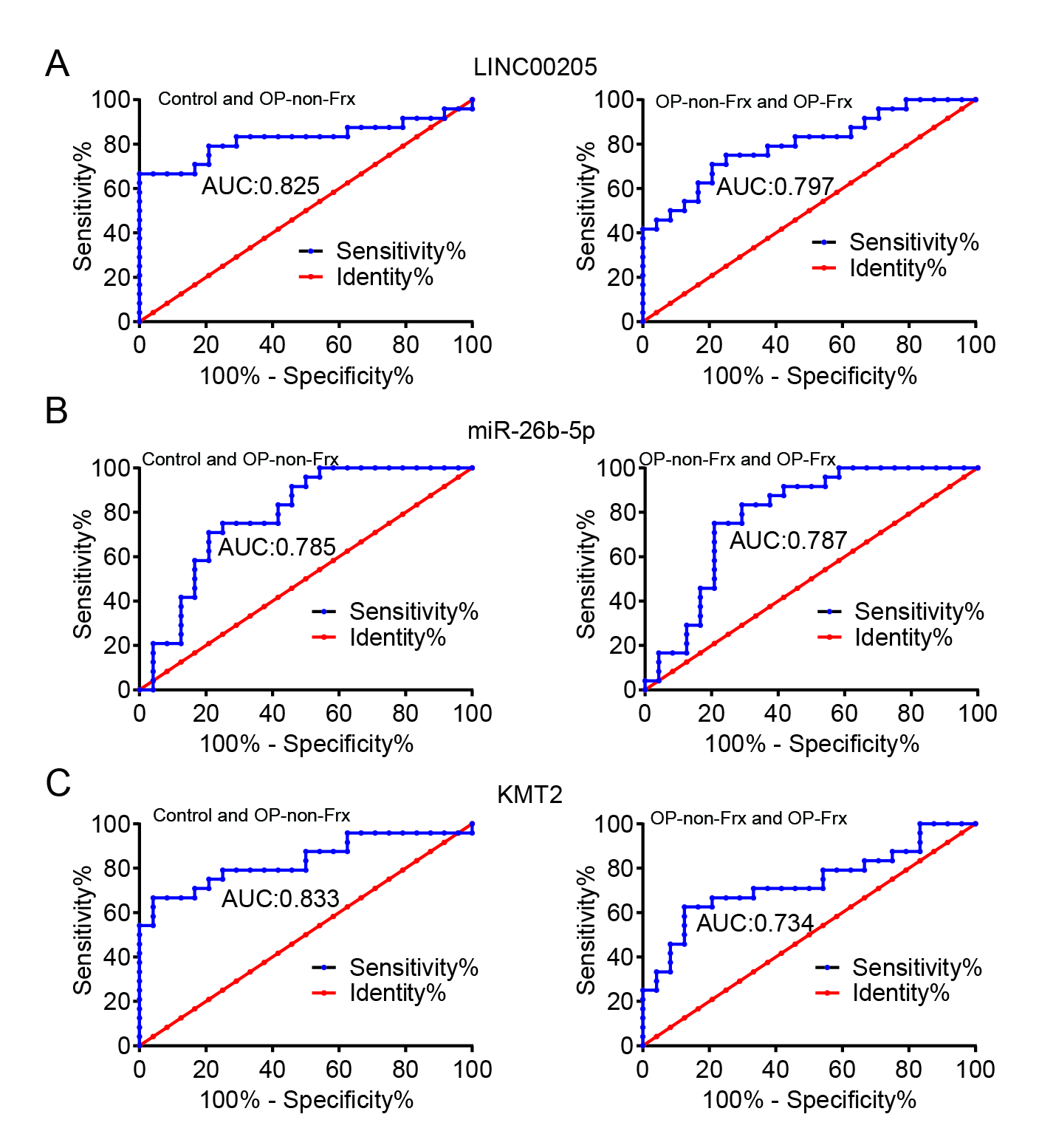

Supplement: Supplementary file 2 — Additional file 2: Supplementary Figure 1 [file 12891_2023_6136_MOESM2_ESM.jpg]

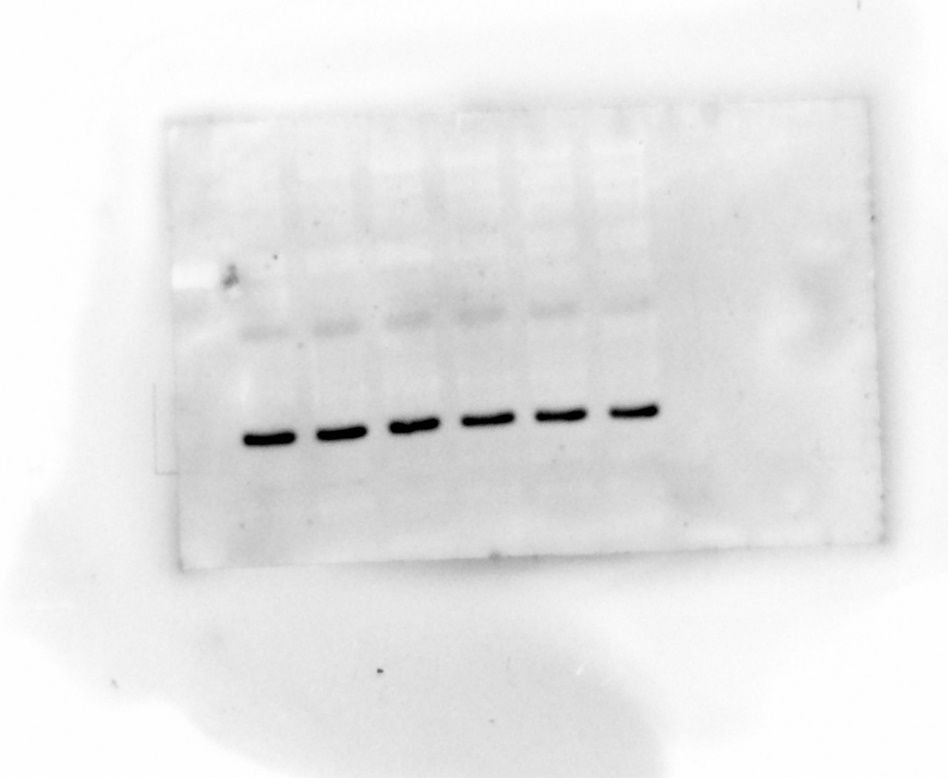


GAPDH


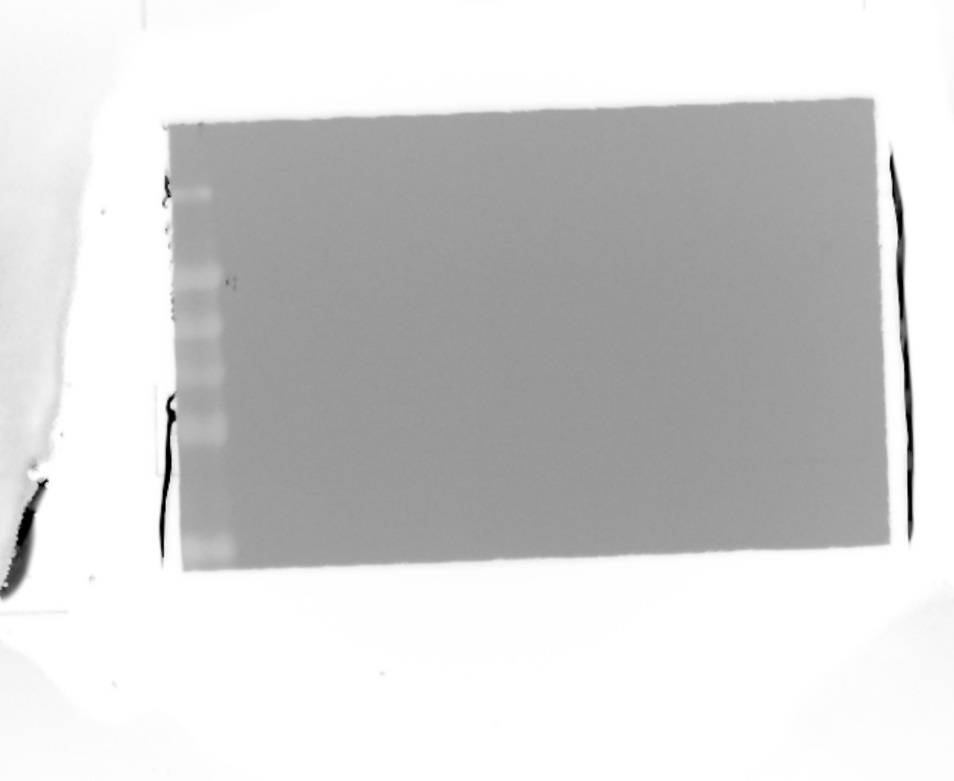


GAPDH-bright field


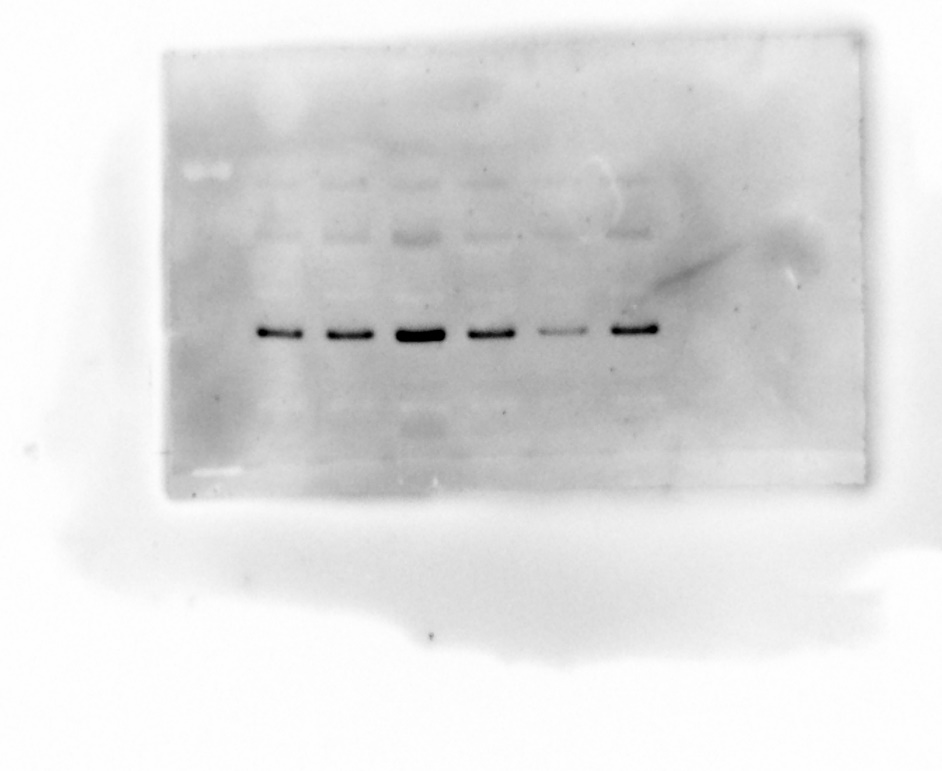


KMT2C


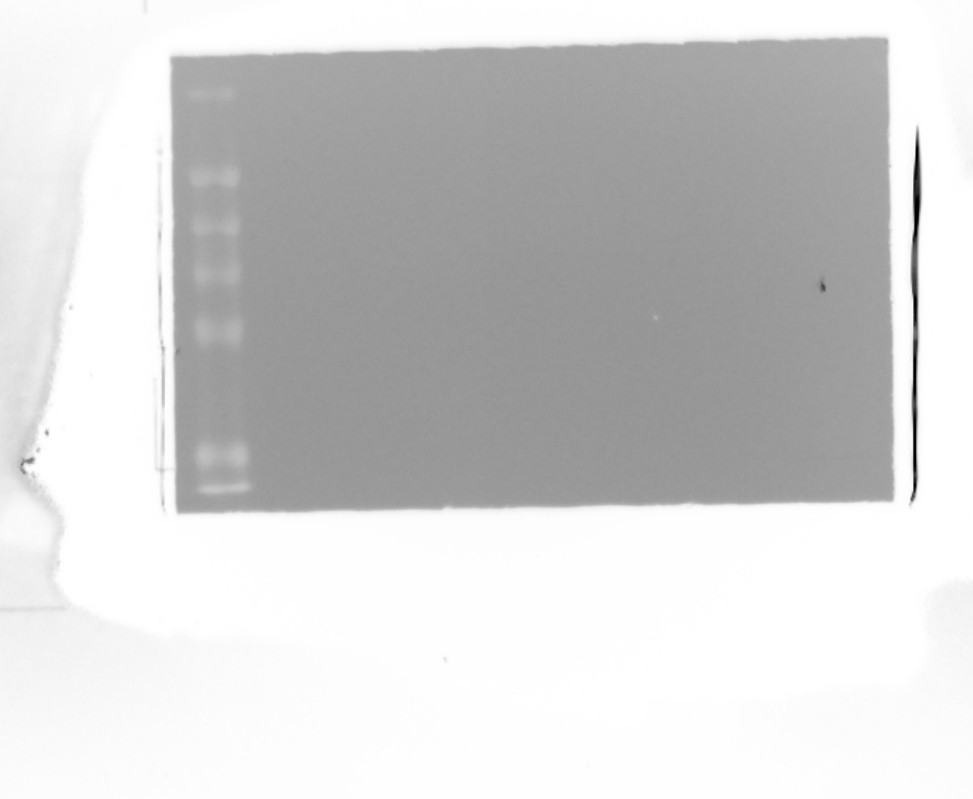


KMT2C-bright field

Supplement: Supplementary file 3 — Additional file 3. [file 12891_2023_6136_MOESM3_ESM.docx]
